# A Miniaturized FBG Tactile Sensor for the Tip of a Flexible Ureteroscope

**DOI:** 10.3390/s25092807

**Published:** 2025-04-29

**Authors:** Shiyuan Dong, Sen Ma, Tenglong Zhou, Yuyang Lou, Xuanwei Xiong, Keyu Wei, Dong Luo, Jianwei Wu, Huanhuan Liu, Ran Tao, Tianyu Yang, Yuming Dong

**Affiliations:** 1 Opto-Electronic Engineering and Technology, Shenzhen Institute of Advanced Technology, Chinese Academy of Sciences, Shenzhen 518055, China; 17864295723@163.com (S.D.); 15961993291@163.com (T.Z.); xw.xiong@siat.ac.cn (X.X.); h51516561@163.com (K.W.); dong.luo@siat.ac.cn (D.L.); hh.liu2@siat.ac.cn (H.L.); ym.dong@siat.ac.cn (Y.D.); 2 School of Physics and Electronic Engineering, Chongqing Normal University, Chongqing 401331, China; jwwu@cqnu.edu.cn; 3 Ekova Technology Co., Ltd., Shenzhen 851000, China; sen.ma@tmeas.cn; 4 Key Laboratory of Optoelectronic Technology and Systems, Chongqing University, Chongqing 400044, China; yy.lou@stu.cqu.edu.cn; 5 Department of Urology, The First Affiliated Hospital of Shenzhen University, Shenzhen Second People’s Hospital, Shenzhen 518055, China

**Keywords:** fiber Bragg grating, tactile sensor, flexible ureteroscopy, force feedback

## Abstract

This work introduces a novel fiber Bragg grating (FBG)-based tactile sensor specifically developed for real-time force monitoring at the tips of flexible ureteroscopes. With a diameter of only 1.5 mm, the sensor features a dual-FBG configuration that effectively separates temperature effects from force signals, integrated with an innovative elastomer structure based on staggered parallelogram elements. Finite element analyses comparing traditional spiral and parallel groove designs indicate that the new configuration not only enhances axial sensitivity through optimized deformation characteristics but also significantly improves resistance to transverse forces via superior stress distribution and structural stability. In the sensor, a suspended lateral FBG is employed for thermal compensation, while an axially constrained FBG is dedicated to force detection. Calibration using a segmented approach yielded dual-range sensitivities of approximately 283.85 pm/N for the 0–0.5 N range and 258.57 pm/N for the 0.5–1 N range, with a maximum error of 0.07 N. Ex vivo ureteroscopy simulations further demonstrated the sensor’s capability to detect tissue–instrument interactions and to discriminate contact events effectively. This miniaturized solution offers a promising approach to achieving precise force feedback in endoscopic procedures while conforming to the dimensional constraints of standard ureteroscopes.

## 1. Introduction

Flexible ureteroscopy (FURS) is a minimally invasive technique for diagnosing and treating upper urinary tract diseases such as kidney stones and urothelial carcinoma by passing through the natural urinary tract, bladder, and ureter [1,2]. Compared to traditional open surgery, FURS offers less pain and faster recovery. However, the complexity of the surgery is increased as urologists need to stand for long periods while holding the flexible ureteroscope for intricate procedures, and the frequent use of X-ray imaging during the operation exposes them to radiation risks [3]. Robotic-assisted FURS systems have emerged to address these challenges [4,5]. Robotic systems not only enable the remote operation of the ureteroscope, reducing radiation exposure, but also improve surgical precision through stable operation [6]. However, traditional robotic-assisted FURS systems in operation lack force feedback at the ureteroscope tip, which makes it difficult for surgeons to perceive tactile information in real-time, such as determining whether the tip is touching stones or normal tissues. This lack of force feedback can lead to instrument damage or surgical complications [7,8]. Therefore, developing a force sensor that can be integrated at the ureteroscope tip is essential.

Force sensors provide real-time force feedback [9]. Currently, there is still a lack of force feedback at the tip of the ureteroscope, which hinders the surgeon’s ability to precisely control the instrument and increases the risk of unintended tissue damage, making the procedure less safe and effective. Existing force sensors can be broadly categorized into electrical-based and optical-based force sensors. Electrical-based sensors, while generally cost-effective and easy to manufacture, suffer from issues like large size and susceptibility to electromagnetic interference. In contrast, optical-based sensors are immune to electromagnetic interference and can be miniaturized, but they are often more complex and costly to implement. Electrical-based force sensors, such as piezoresistive and piezoelectric types, are limited by their large size, which makes them incompatible with ureteroscopes. Additionally, they are unable to resist electromagnetic interference, significantly restricting their application [10]. Optical-based force sensors are mainly classified into intensity-modulated, phase-modulated, and wavelength-modulated types. Intensity-modulated fiber-optic force sensors rely on the measurement of voltage or current induced by force-induced intensity changes [11]. However, each fiber-optic sensor requires two independent optical fibers to reflect the interaction forces, which negatively impacts miniaturization and makes integration at the ureteroscope tip challenging. Phase modulation employs Fabry–Perot interferometry for measurements [12,13], which involves using multiple reflections between two parallel reflective surfaces to create interference patterns that can be used to determine changes in distance or force. However, the maximum measurement range is limited to half the wavelength of the light, which cannot meet the high precision force measurement requirements at the ureteroscope tip. Wavelength modulation, using fiber with inscribed fiber Bragg gratings (FBGs) as sensing elements, can overcome the limitations of optical intensity fluctuation and phase discontinuity [14]. Additionally, FBG-based wavelength modulation provides high sensitivity, stability, and ease of integration, making it a superior choice for precise force measurements at the ureteroscope tip. Recently, Zhao et al. [15] presented embedded FBG sensors fabricated by ultrasonic additive manufacturing, demonstrating robust high-frequency dynamic strain measurements in metal structures. In comparison, our sensor features significant miniaturization, tailored structural optimization, and comprehensive calibration methods, specifically designed for delicate force measurements in ureteroscopic surgical applications.

FBG technology, due to its high sensitivity, compact size, and excellent biocompatibility, has emerged as an ideal solution for sensing tactile information at the tip of ureteroscopes [16]. FBG-based force sensors can be integrated into the tip of flexible ureteroscopes to detect forces applied at the tip by measuring variations in the Bragg wavelength. By providing real-time force feedback, these sensors significantly enhance the safety of surgical procedures and the operational precision of the surgeon [17]. Gan et al. [18] developed a uniaxial FBG force sensor for palpation in minimally invasive surgeries, incorporating an elastomer with a helical structure to improve force sensitivity. However, this helical structure is susceptible to interference from lateral forces, limiting its practical utility. Gao et al. [19] integrated an FBG force sensor into the forceps used in retinal microsurgery, but the sensor’s low accuracy makes it unsuitable for detecting subtle contact forces in FURS applications. He et al. [20] developed an FBG tactile sensor for vitreoretinal surgery and discussed the influence of different structures on force sensing. However, this sensor has a limited force detection range, while FURS generally requires a force detection range of up to 1 N. In our previous work [21], a dual-elastomer FBG tactile sensor was developed for temperature decoupling. However, the 3 mm diameter of the sensor limits its integration into the ureteroscope tip.

To address these limitations, this paper proposes a miniature FBG tactile sensor with resistance to lateral forces and temperature monitoring capabilities. The sensor is integrated into the tip of a flexible ureteroscope to measure contact forces applied to the ureteral wall or kidney stones. Section 2 details the sensor’s structural design and sensing principle and analyzes finite element analysis comparing various structures to demonstrate the sensor’s resistance to lateral interference. Section 3 presents the fabrication and calibration of the sensor prototype and includes integration of the sensor into the ureteroscope tip for performance testing in a simulated lithotripsy procedure, detailing the testing environment, equipment used, and specific metrics evaluated.

## 2. Sensor Design

### 2.1. Force Sensor Design

**Figure 1:**
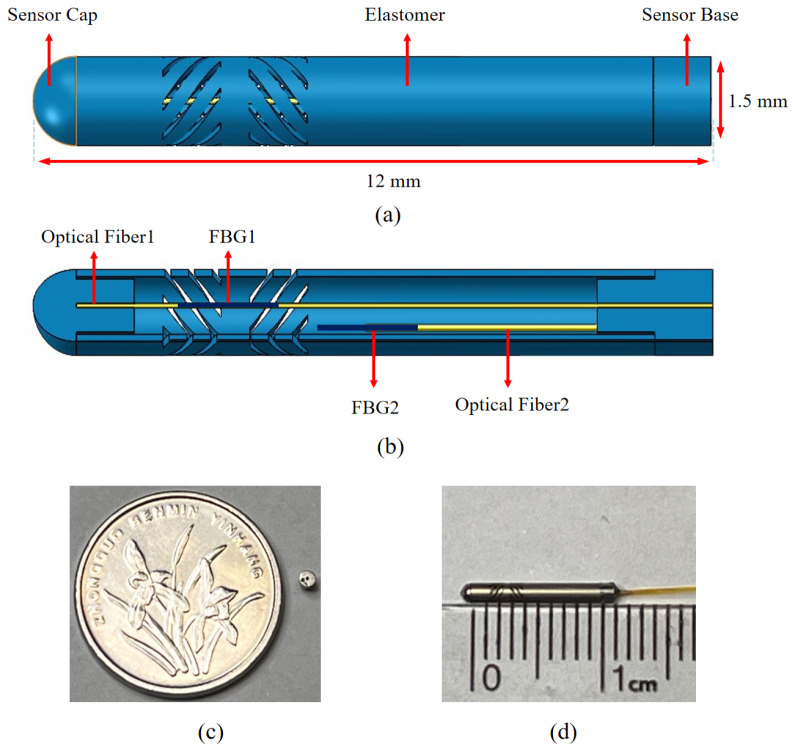
Schematic diagram of designed sensor for flexible ureteroscope. (**a**) Structure and assembly overview; (**b**) crosssection; (**c**) sensor base; (**d**) prototype.

The designed haptic sensor should match the size of the working channel of FURS and be capable of detecting forces within the range of 0.1 N to 1 N typically encountered during procedures. This is crucial to ensure adequate maneuverability and precise force feedback, which are essential for effective FURS performance. Typically, the diameter of a force sensor in FURS should range from 1.3 mm to 2 mm, with an axial force measurement range of up to 1 N. As shown in Figure 1a, a miniature FBG haptic sensor was designed to meet the requirements of a ureteroscope [22]. With a diameter of only 1.5 mm, it is the smallest reported FBG haptic sensor, capable of meeting the specifications of the ureteroscope working channel.

Having the smallest diameter is advantageous as it allows for easier insertion and navigation within the narrow ureteroscope working channel, minimizing potential interference and enhancing maneuverability. In comparison, typical FBG sensors used in similar applications have diameters ranging from 2 mm to 3 mm, making this design substantially better suited for minimally invasive procedures. The haptic sensor consists of a sensor cap, an elastomer, a sensor base, and two optical fibers, each embedded with an FBG. Figure 1b shows the cross-sectional view of the sensor. The ends of the central axial fiber are bonded within fiber holes designed into the sensor cap and sensor base, with FBG1 being used to sense strain induced by both temperature and force. The elastomer segment with the curved grooves was custom-modeled in SolidWorks2021 and then fabricated via laser machining at Xinpeng, using 304 stainless steel for its biocompatibility and mechanical robustness. FBG1 distinguishes between temperature and force-induced strain through a calibration process that compensates for temperature effects, which involves applying a reference load under controlled temperature conditions to develop a correction factor. This calibration allows precise force measurements unaffected by temperature variations. Figure 1c,d show the prototype of the assembled sensor. A pre-tension of 0.5 N was applied to the fiber before bonding to ensure a linear response to strain, which is crucial for maintaining measurement accuracy and reliability, as it allows for consistent and predictable sensor behavior under varying strain conditions. To ensure stable bonding and prevent the fiber from loosening under the maintained 0.5 N pre-tension, we used Loctite 4011 instant adhesive and allowed each bonding step to cure for 24 h before proceeding with further assembly. Compared with the traditional method of directly applying glue to the FBG, this approach avoided uneven glue thickness, thereby mitigating chirp effects, which are variations in the reflected wavelength spectrum caused by uneven strain distribution. The front end of the lateral fiber was suspended within the elastomer, with FBG2 used to decouple temperature effects from the axial fiber. The sensitivity-enhancing structure of the elastomer was designed in an alternating staggered parallelogram configuration. This structure enhances sensitivity by optimizing stress distribution and reducing localized strain concentrations, providing a more uniform response compared to conventional helical or parallel rectangular groove structures. Compared with conventional helical or parallel rectangular groove structures, this design increases the sensor’s resistance to transverse forces. The materials used in the designed FBG haptic sensor are 304 stainless steel, which provides excellent biocompatibility and mechanical robustness, making it suitable as a substrate material for sensors in FURS.

### 2.2. Flexure Design for Instrument Shaft

**Figure 2:**
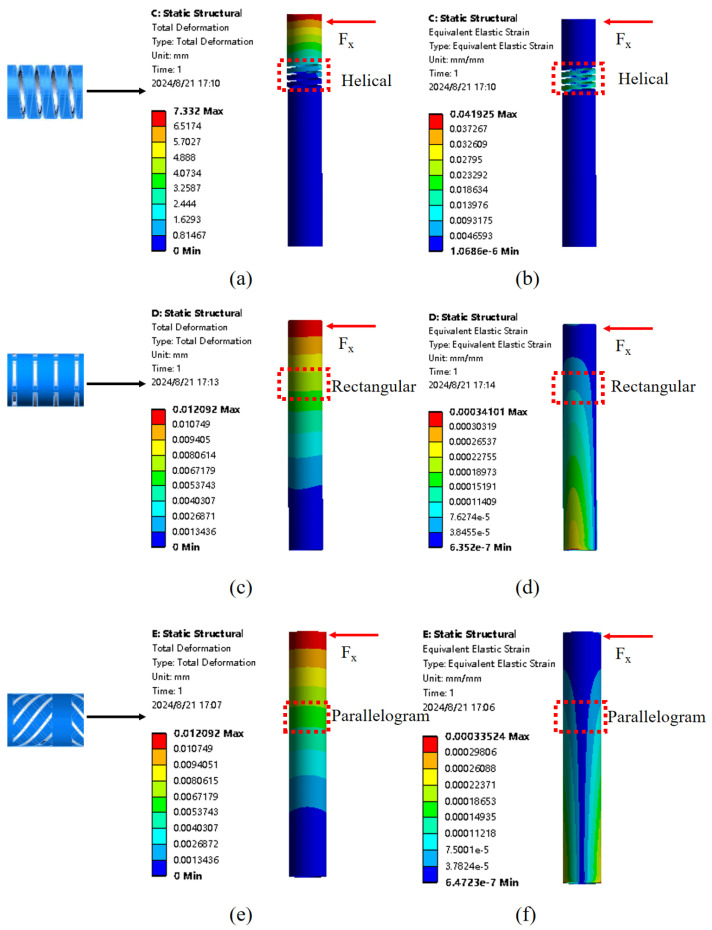
Under a 1 N lateral force load: (**a**) The total deformation of the helical structure. (**b**) The total strain of the helical structure. (**c**) The total deformation of the parallel rectangular groove structure. (**d**) The total strain of the parallel rectangular groove structure. (**e**) The total deformation of the staggered parallelogram structure. (**f**) The total strain of the staggered parallelogram structure.

In the design of elastomer sensitivity-enhancing structures, resistance to transverse forces has often been overlooked, which is crucial for ensuring stability and accurate force detection in multiple directions. This has significantly limited the FBG haptic sensor in FURS to detecting forces only in the vertical direction, thereby reducing its overall applicability. Helical structures, parallel rectangular groove structures, and alternating staggered parallelogram structures were designed to evaluate their effectiveness in resisting transverse forces in the FBG haptic sensor. This comparison aims to identify the most effective design in enhancing the overall performance of the sensor, particularly in resisting transverse forces while maintaining sensitivity. The helical structure incorporates a spiral design in the flexural region of the elastomer to improve sensitivity to axial forces. However, the hollowed-out elastomer of the helical structure renders it ineffective in resisting transverse forces, as was verified in simulations detailed later. The traditional parallel rectangular groove structure enhances sensitivity by concentrating strain in specific regions of the elastomer, thereby improving the response of the FBG sensor. Compared to the helical structure, this design offers some resistance to transverse forces; however, its performance remains suboptimal due to insufficient stiffness and limited ability to uniformly distribute stress, thereby hindering its overall effectiveness. Additionally, the close spacing of adjacent rectangular grooves complicates the manufacturing process, thus increasing the complexity of sensor fabrication. To alleviate these difficulties, increasing the spacing between grooves or using a staggered groove arrangement may simplify the manufacturing process while maintaining sensitivity. However, increasing the spacing could reduce the concentration of strain in specific areas, potentially affecting sensitivity, whereas a staggered arrangement may introduce complexity in maintaining uniform groove alignment. Figure 2e,f depict the newly proposed alternating staggered parallelogram structure, designed to improve performance. The parallelogram design enhances force sensitivity, while the alternating arrangement on the upper and lower sides mitigates deformation resulting from transverse forces, thereby improving overall sensor reliability. Compared to the helical and rectangular groove designs, the parallelogram structure demonstrates superior performance regarding both sensitivity and resistance to transverse forces. This design is also straightforward to process, readily manufacturable, and highly robust, making it an attractive choice for practical applications.

Finite element simulation analysis was conducted on the three structures to determine their resistance to transverse forces. A lateral force of 1 N was applied to each structure to observe their deformation and strain, thus determining the final design of the force sensor. As shown in Figure 2a,c,e, the overall deformation diagrams of the helical, parallel rectangular groove, and staggered parallelogram structures under a 1 N lateral force are shown, respectively. Similarly, Figure 2b,d,f depict the overall strain diagrams for these three structures. Significant deformation and strain were observed in the sensitivity-enhancing structures. The helical structure exhibited much greater deformation and strain compared to the other two structures, indicating its inability to effectively resist transverse forces, presenting significant limitations in FURS. Consequently, the helical structure was not selected as the sensitivity-enhancing structure. Although the traditional rectangular groove structure demonstrated reduced deformation and strain compared to the helical structure, it was difficult to manufacture, demanded high precision, and lacked effectiveness in resisting transverse forces compared to the third structure. The staggered parallelogram structure exhibited robust resistance to transverse force-induced errors and was comparatively simpler to fabricate. Thus, the staggered parallelogram structure was adopted as the sensitivity-enhancing component in the sensor design.

### 2.3. Force Calculation Algorithm

The stiffness of the designed sensor structure includes three key stiffness elements, the stiffness of the circular hinge, $K_{S}$, the overall stiffness of the central axial fiber, $K_{O}$, and the stiffness of the beam structure, $K_{B}$, which can be represented as follows:

(1) $$\begin{array}{c} K_{S}=\frac{E_{S}I_{S}}{L_{S}} \end{array}$$

(2) $$\begin{array}{c} K_{O}=\frac{E_{O}A_{O}}{L_{O}} \end{array}$$

(3) $$\begin{array}{c} K_{B}=\frac{E_{B}I_{B}}{L_{B}} \end{array}$$

where $E_{S}$, $E_{O}$, and $E_{B}$ are the elastic moduli of the circular hinge, central axial fiber, and beam structure, respectively; $I_{S}$ and $I_{B}$ are the moments of inertia for the cross-sections of the circular hinge and beam, respectively; $A_{O}$ is the effective cross-sectional area of the central axial fiber; and $L_{S}$, $L_{O}$, and $L_{B}$ are the lengths of the circular hinge, central axial fiber, and beam, respectively. Combining these stiffness elements, the total equivalent stiffnes $K_{eq}$ of the designed FBG tactile sensor can be further expressed as

(4) $$\begin{array}{c} K_{eq}={\left(\frac{1}{K_{S}}+\frac{1}{K_{O}}+\frac{1}{K_{B}}\right)}^{-1} \end{array}$$

When the FBG tactile sensor is subjected to an axial force, $F_{Z}$, it causes a shift in the Bragg central wavelength of FBG1. This relationship can be described using the elasto-optic effect, expressed as

(5) $$\begin{array}{c} \Delta \lambda_{1}=\lambda_{1}\left[\left(1-\rho_{e}\right)\frac{F_{Z}}{K_{eq}A_{FBG}}\right] \end{array}$$

where $\Delta \lambda_{1}$ represents the wavelength shift in FBG1, $\lambda_{1}$ is the initial central wavelength of FBG1, $\rho_{e}$ is the effective elasto-optic coefficient of FBG1, and $A_{FBG}$ is the cross-sectional area of FBG1. In addition to force affecting the wavelength shift of FBG1, temperature also induces a wavelength shift. The temperature-induced wavelength shift $\Delta \lambda_{T}$ of FBG1 can be expressed as

(6) $$\begin{array}{c} \Delta \lambda_{T}=\lambda_{1}\left(\alpha_{f}+\zeta_{f}\right)\Delta T \end{array}$$

where $\alpha_{f}$ is the thermal expansion coefficient of the fiber, $\zeta_{f}$ is the thermo-optic coefficient of the fiber, and ΔT is the temperature change. Considering the simultaneous effects of temperature and axial force, the total wavelength shift $\Delta \lambda_{total}$ of FBG1 can be expressed as

(7) $$\begin{array}{c} \Delta \lambda_{total}=\Delta \lambda_{1}+\Delta \lambda_{T} \end{array}$$

Since the front end of FBG2 is suspended inside the elastomer and is not bonded to the sensor cap, it is only affected by temperature-induced wavelength shift $\Delta \lambda_{T2}$, which can be expressed as

(8) $$\begin{array}{c} \Delta \lambda_{T_{2}}=\lambda_{2}\left(\alpha_{f}+\zeta_{f}\right)\Delta T \end{array}$$

where $\lambda_{2}$ is the Bragg central wavelength of FBG2. By using FBG2, the cross-interference effect between force and temperature in FBG1 can be decoupled. The decoupled axial force $F_{Z}$ can be expressed as

(9) $$\begin{array}{c} F_{Z}=\frac{\left(\Delta \lambda_{total}-\frac{\Delta \lambda_{T2}\lambda_{1}}{\lambda_{2}}\right)K_{eq}A_{FBG}}{\lambda_{1}\left(1-\rho_{e}\right)} \end{array}$$

Thus, it can be seen that the designed FBG force sensor can accurately measure axial force while effectively avoiding the influence of temperature.

### 2.4. Static Finite Element Simulation

**Figure 3:**
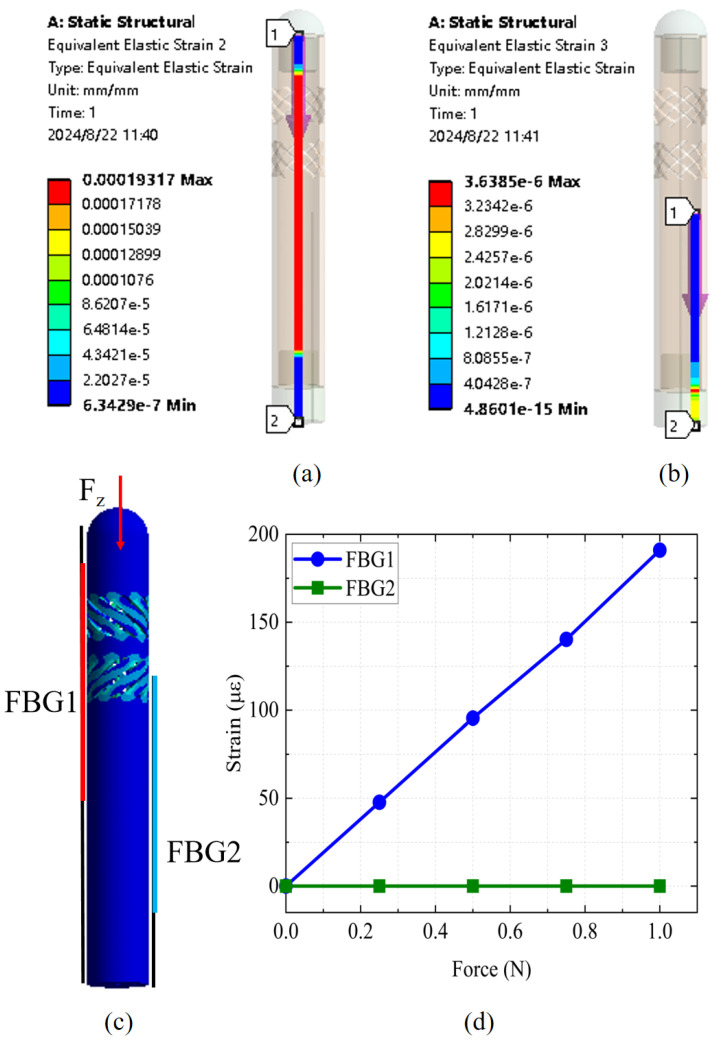
(**a**) The strain distribution of the central axial fiber under a 1 N axial force. (**b**) The strain distribution of the lateral fiber under a 1 N axial force. (**c**) The overall strain distribution of the sensor under axial force. (**d**) The response of FBG1 and FBG2 to axial force.

Finite element simulation analysis was conducted on the designed FBG tactile sensor to predict its performance. First, axial force simulation analysis was performed on the sensor. Figure 3a,b show the strain generated in the two fibers of the FBG tactile sensor when subjected to an axial force of 1 N. It can be observed that the central axial fiber exhibited significant strain under axial force, demonstrating high sensitivity to axial forces. In contrast, the lateral fiber, which was suspended inside the elastomer, did not produce strain even when the sensor was subjected to axial force.

Figure 3c shows the overall strain distribution of the sensor under axial force, with significant strain occurring in the flexure region, demonstrating the effectiveness of the sensitivity-enhancing design. Axial force simulations were conducted four times in the range of 0–1 N, with a step size of 0.25 N, with the strain results for FBG1 and FBG2 illustrated in Figure 3d. FBG1 demonstrated high sensitivity and linearity, while FBG2 remained unaffected by the applied force. The static simulation of axial force confirmed the effectiveness and feasibility of the FBG tactile sensor design for axial force detection. Because the designed FBG sensor geometry strongly resists lateral deformation, any angled load primarily induces axial strain in the sensing region. Consequently, even if the force has both axial and lateral components, the measured strain remains dominated by the axial portion, ensuring that the overall reading remains both meaningful and clinically relevant.

**Figure 4:**
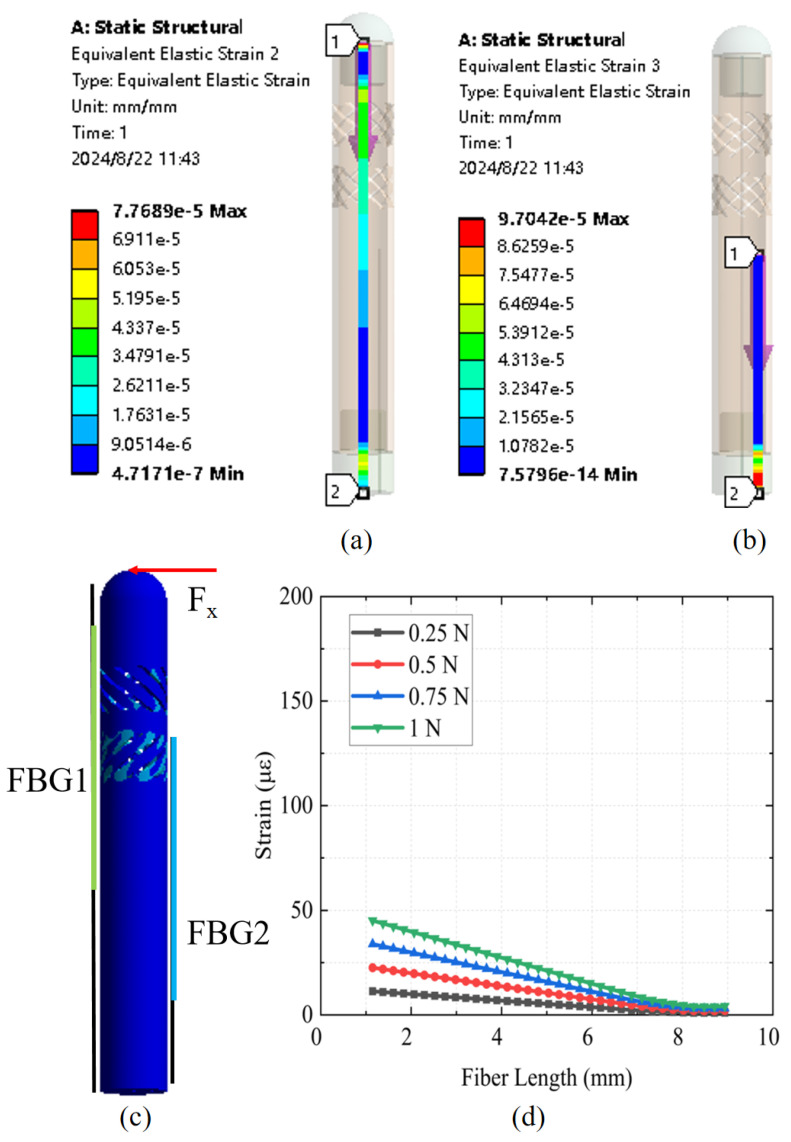
(**a**) Strain distribution of the central axial fiber under a 1 N lateral force; (**b**) Strain distribution of the lateral fiber under a 1 N lateral force; (**c**) Overall strain distribution of the sensor under lateral force; (**d**) Response of FBG1 to lateral force.

To verify the resistance of the designed FBG tactile sensor to lateral forces, static simulation analysis was conducted under the application of a lateral force. Figure 4a,b present the strain distribution of the central axial fiber and the lateral fiber when the sensor was subjected to a 1 N lateral force. It can be observed that, under the influence of lateral force, the central axial fiber experienced minimal strain compared to that under axial force, demonstrating a significant sensitivity difference between axial and lateral forces in the designed structure. The lateral fiber remained insensitive to the applied force, exhibiting negligible strain. Figure 4c illustrates the overall strain distribution of the sensor under lateral force; compared to in Figure 3c, only minimal strain is observed in minor flexure regions. Figure 4d depicts the strain distribution of the central axial fiber under four different lateral forces. Compared to the strain generated under axial force, the strain induced by lateral force was significantly smaller. In summary, the designed force sensor exhibited high sensitivity and linearity to axial force while minimizing sensitivity to lateral forces. Additionally, the lateral fiber’s lack of sensitivity to applied forces further confirms the sensor’s ability to decouple temperature effects.

## 3. Sensor Experiments

### 3.1. Sensor Parts

During the fabrication process of the designed FBG tactile sensor, the initial step involved customizing the optical fiber inscribed with FBG. To ensure the alignment of the FBG region with the flexure section of the elastomer, a black marker was applied near the FBG during the design stage to facilitate fiber bonding. The FBG was fabricated using femtosecond laser inscription, a technique that provides precise control over grating parameters while minimizing thermal damage. The grating exhibits a physical length of 2 mm, a central wavelength of 1550 nm, and a reflectivity greater than 90%. These parameters were deliberately selected to meet the rigorous integration requirements of a flexible ureteroscope.

Specifically, the 2 mm grating length allows the sensing element to be seamlessly incorporated within the 1.5 mm working channel, ensuring that the overall sensor remains compact and does not impede maneuverability. The choice of a 1550 nm wavelength is advantageous due to its compatibility with standard optical interrogation systems and its low transmission loss in optical fibers, which is vital for accurate sensing in a clinical environment. Furthermore, the high reflectivity ensures a robust signal-to-noise ratio, enabling the precise detection of minor wavelength shifts induced by axial forces and temperature fluctuations. Regarding manufacturing, given that the designed FBG tactile sensor is intended for FURS applications with a diameter of only 1.5 mm, stringent requirements are imposed due to the small scale. The sensor is constructed from medical-grade 304 stainless steel, with components fabricated using a Swiss-type lathe in a single-step machining process to ensure high precision. Furthermore, for the elastomer component, since the hollow cylindrical body has a wall thickness of only 0.3 mm, laser cutting technology was employed to create a sensitivity-enhancing structure.

### 3.2. Sensitivity Coefficients Calibration

The designed FBG tactile sensor was calibrated for force sensitivity and temperature sensitivity coefficients. First, a fixture was designed to secure the sensor, which depicted the model and the actual image of the fixture, respectively. The fixture consisted of an upper and a lower part, with a 1.5 mm through-hole for placing the designed FBG tactile sensor, which was then secured using screws.

The calibration system mainly consisted of the designed sensor, an ATI 6-axis force-torque sensor (Nano17 SI-12-0.12, Apex, NC, USA), a NetBox (9105-NETBA, ATI, Apex, NC, USA) used for data transfer, an FBG interrogator (SA-10002454; sampling rate: 100 Hz; resolution: 1 pm, Shenzhen, China), a automatic displacement stage, a manual dispalcement stage, a fixture, and a computer. During the calibration process, since movement in the automated translation stage was required, the force value monitored by the ATI sensor and the central wavelength shift displayed by the FBG interrogator were recorded simultaneously. Therefore, a LabVIEW program was developed. Movement in the x-direction of the automated translation stage corresponded to the z-direction of the FBG sensor. Thus, the control page for the x-direction of the automated translation stage was configured within the software interface. The real-time force feedback from the ATI sensor and the central wavelength value of the FBG interrogator were also displayed on the main interface of the software. By pressing and holding the save button, the force values from the ATI sensor and the wavelength shifts from the FBG interrogator could be saved simultaneously, facilitating the force calibration experiment.

**Figure 5:**
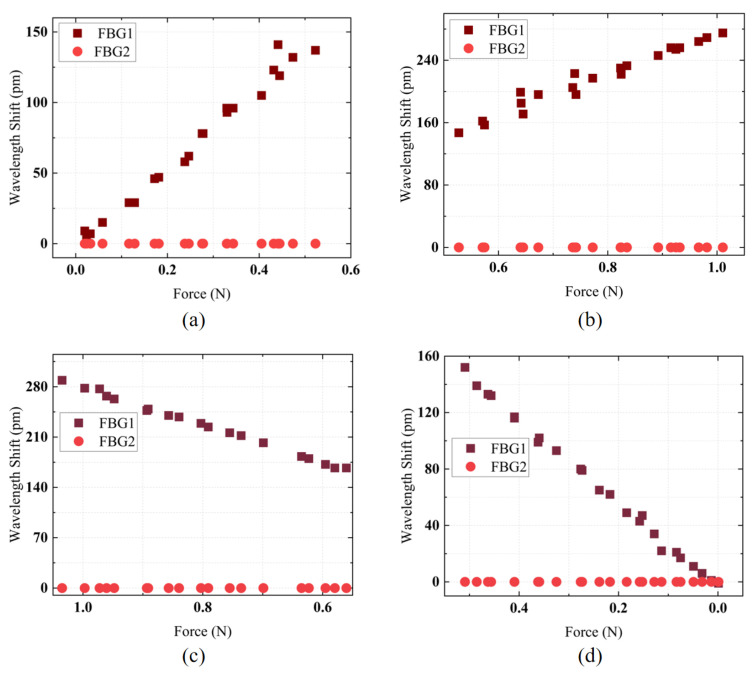
Force calibration data: (**a**) 0–0.5 N; (**b**) 0.5–1 N; (**c**) 1–0.5 N; (**d**) 0.5–0 N.

The automated translation stage was moved to allow the ATI sensor to make contact with the FBG sensor for calibration. Data recording was performed using the designed calibration software. Four experiments were conducted for the intervals [0–0.5 N], [0.5–1 N], [1–0.5 N], and [0.5–0 N] to record the sensitivity of the designed FBG tactile sensor in these ranges. The experimental results are shown in Figure 5a–d. In the [0–0.5 N] range, the FBG tactile sensor exhibited a sensitivity of 283.85 pm/N with a linearity of 98.27%; in the [0.5–1 N] range, the sensitivity was 258.57 pm/N with a linearity of 96.34%; in the [1–0.5 N] range, the sensitivity was 260.75 pm/N with a linearity of 99.68%; and in the [0.5–0 N] range, the sensitivity was 296.44 pm/N with a linearity of 99.67%. Because of the 0.5 N pre-tension in the optical fiber, the elastomeric structure initially deformed more readily at lower loads, yielding higher sensitivity; as the load surpassed 0.5 N, the elastomer reached a stiffer regime with slightly reduced sensitivity. Nevertheless, each sub-range remained linear due to the consistent mechanical properties of the elastomer and uniform fiber bonding. This slight discrepancy arose from minor mechanical hysteresis in the elastomeric structure and adhesive bond, where progressive elastic deformation upon loading and a brief relaxation delay upon unloading led to small differences. The calibration results demonstrate that the designed force sensor has high force sensitivity and linearity, ensuring high accuracy in force detection, which is advantageous for contact force measurement in FURS.

### 3.3. Investigation of Dynamic Performance

**Figure 6:**
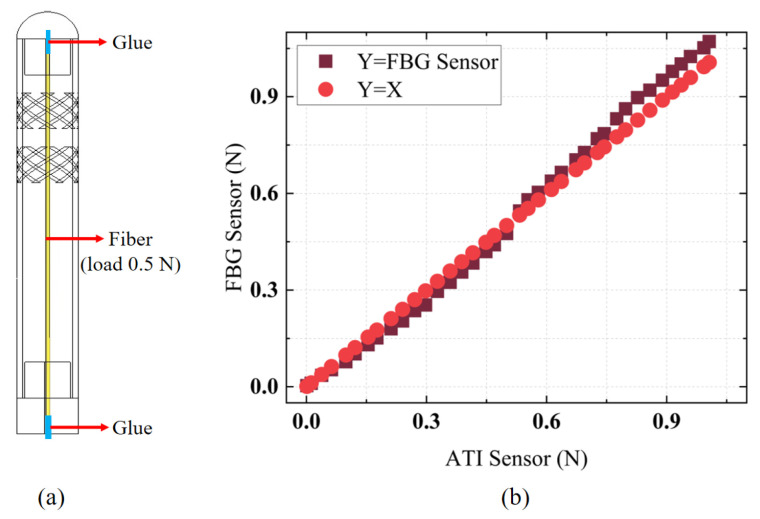
(**a**) The internal packaging structure of the sensor. (**b**) The dynamic response experiment using the designed force sensor.

The designed FBG tactile sensor was calibrated using a segmented calibration method within small force ranges to facilitate accurate contact force measurements in practical applications. The higher sensitivity in the [0–0.5 N] range compared to the [0.5–1 N] range was attributed to the pre-tension of 0.5 N applied to the fiber before bonding the FBG tactile sensor, as shown in Figure 6a, to maintain high force sensitivity and linearity. Thus, the sensitivity in the [0–0.5 N] range was greater than that in the [0.5–1 N] range. Additionally, the segmented calibration method improved force detection accuracy in practical scenarios. A dynamic force load was applied to the FBG tactile sensor to validate the accuracy of its force measurements. Figure 6b presents a comparison between the force measurements obtained from the FBG tactile sensor and the ATI force sensor. It is evident that, within the dynamic force range of [0–1 N], the error obtained through segmented calibration did not exceed 0.07 N, and the sensor exhibited a high linear response, allowing for accurate contact force measurements.

### 3.4. Investigation of Dynamic Performance

**Figure 7:**
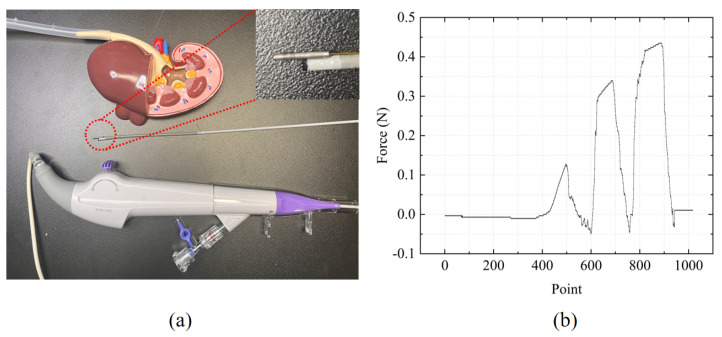
(**a**) A flexible ureteroscope with a force sensor prototype. (**b**) Force responses at the tip of the ureteroscope.

In FURS, the surgeon first inserts the flexible ureteroscope into the patient’s urethra, then advances it into the bladder. Subsequently, it is navigated from the bladder into the ureter. Due to the narrowness of the ureter, the surgeon must carefully manipulate the ureteroscope to avoid damaging the surrounding tissue. Finally, the ureteroscope is advanced into the renal pelvis and calyx regions of the kidney. In lithotripsy, stones are typically located in the ureter and kidney. Without force-sensing capabilities at the ureteroscope tip, excessive contact force during ureter insertion may damage the tissue, and distinguishing between stones and normal tissue is also challenging. The designed FBG tactile sensor was integrated into the tip of the flexible ureteroscope, and a kidney model was used to simulate the insertion process of the ureteroscope during lithotripsy, as shown in Figure 7a. The ureter was fabricated using 3D printing, with some protrusions designed on the inner wall to simulate the presence of stones. The force measurement results are shown in Figure 7b. The first peak represents the force response when contacting the ureter wall, while the subsequent two peaks represent the force response when touching the stones. It can be observed that the contact force was larger when touching the stones, whereas it was smaller in other regions. This verifies that the designed FBG tactile sensor in FURS not only has the capability to differentiate between stones and normal tissue but also protects the ureter wall from damage caused by medical instruments.

## 4. Conclusions

This work developed an FBG micro-force sensor that can be integrated into the tip of a flexible ureteroscope, with the objective of detecting and distinguishing stones from tissues during FURS lithotripsy procedures. First, the designed sensor has a total diameter of only 1.5 mm, exceeding the size constraints of current force sensors. Second, a flexure structure was designed to resist transverse forces, to enhance axial force sensitivity and mitigate lateral force load interference during force measurements. Comparative modeling and simulation analyses were conducted with several existing sensitivity-enhancing structures, demonstrating the superior performance of the proposed structure. Finally, an experimental calibration platform was established to determine the accuracy and sensitivity of the designed force sensor. Additionally, a simulated lithotripsy experiment was conducted. The sensor was integrated into the ureteroscope tip to conduct object detection experiments. Experimental results showed that the sensor could distinguish between various objects at the ureteroscope tip. The study of the flexible ureteroscope tip based on the FBG force sensor shows significant potential for improving the safety and efficacy of surgical interventions. By integrating advanced fiber-optic sensing technology, robotics, and artificial intelligence, real-time force feedback during surgery enhances the surgeon’s tactile perception and minimizes surgical risks. Future research will further advance the development of multimodal sensing technology, machine learning, and miniaturization, fostering innovation and breakthroughs in the application of FURS.

## Data Availability

The experimental data are contained within this article.

## Abbreviations

The following abbreviations are used in this manuscript:

FURS	Flexible ureteroscopy
FBG	Fiber Bragg grating
